# Isolated in the highlands, found in the museum: A new species of *Characidium* (Crenuchidae) from a Bolivian National Park, with a CT scan revealing features

**DOI:** 10.1111/jfb.70426

**Published:** 2026-03-27

**Authors:** Leonardo Oliveira‐Silva, Karina Or, Angela Zanata, Scott A. Schaefer

**Affiliations:** ^1^ Departamento de Biologia Estrutural e Funcional Instituto de Biociências, Universidade Estadual Paulista São Paulo Brazil; ^2^ Department of Ichthyology American Museum of Natural History New York New York USA; ^3^ Museo de Historia Natural Noel Kempff Mercado Universidad Autónoma Gabriel René Moreno Santa Cruz Bolivia; ^4^ Programa de Pós‐Graduação em Biodiversidade e Evolução Instituto de Biologia, Universidade Federal da Bahia Salvador Brazil

**Keywords:** Characidiinae, freshwater fishes, highland rivers, protected areas, Weberian apparatus

## Abstract

A new species of *Characidium* is described from a small, isolated river in the highland areas of Noel Kempff Mercado National Park, Bolivia. The new taxon can be diagnosed by the presence of a relatively broad and conspicuous dark midlateral stripe extending from the tip of snout to the base of the caudal fin, markedly darker than the vertical bars (when present), added to the absence of a distinct humeral blotch. Additional distinguishing features include a complete lateral line, presence of adipose fin, 12 circumpeduncular scale rows, four horizontal scale rows above the lateral line, presence of the parietal branch of the supraorbital canal and a conspicuous basicaudal spot. Some specimens also exhibit reddish‐brown pigmentation on the paired, dorsal and caudal fins, a condition otherwise restricted to few congeners. This discovery highlights the hidden fish diversity of poorly explored highland rivers and protected areas within Neotropical region. In addition, we discuss osteological features of the Weberian apparatus, documented for the first time in the genus using computed tomography (CT) scan imagery. Also, geomorphological processes that may have influenced the isolation of the new species on the Huanchaca Meseta are discussed.

## 1 INTRODUCTION

The genus *Characidium* Reinhardt is the most species‐rich within the subfamily Crenuchinae (Characiformes: Crenuchidae), with 91 valid species widely distributed in South and Central America (Fricke et al., 2026; Oliveira‐Silva et al., 2026; Zanata et al., 2025). The unique morphological synapomorphy proposed for the genus to date is the presence of a basicaudal spot (Buckup, 1993a), a trait that is absent in some species (Oliveira‐Silva et al., 2024). The monophyly of the genus has been questioned by the results of recent molecular research studies (Oliveira et al., 2011; Oliveira‐Silva et al., 2024). Although a comprehensive phylogenetic analysis specifically testing this hypothesis is yet to be done, species richness within the genus has substantially expanded in recent years due to the publication of several new taxa (e.g., Oliveira‐Silva et al., 2026; Stabile et al., 2025; Zanata et al., 2025).

Among the rivers draining through Bolivia into the Madeira River, Amazon basin, the following *Characidium* species have been documented: *Characidium bolivianum* Pearson, *Characidium fasciatum* Reinhardt, *Characidium heinianum* Zarske & Géry, *Characidium laterale* (Boulenger), *Characidium purpuratum* Steindachner, *Characidium schindleri* Zarske & Géry, *Characidium steindachneri* Cope and *Characidium zebra* Eigenmann (Frick et al., 2026). Among these, only three species (i.e., *C. bolivianum*, *C. heinianum* and *C. schindleri*) were originally described from Bolivian waterways, specifically from the Beni and Mamoré rivers, significant tributaries of the Madeira River flowing through Bolivian territory. In addition to these, other significant tributaries of the Iténez‐Guaporé River, also draining through Bolivian territory, include the San Miguel, San Martin, Paraguá and Pauserna rivers. However, ichthyofaunal studies focused on these drainages remain scarce (Sarmiento, 1998). The genus *Characidium* is represented by three species on the Iténez‐Guaporé basin (i.e., *Characidium barbosai* Flausino Junior, Lima, Machado & Melo, *Characidium fleurdelis* Zanata, Oliveira‐Silva & Ohara and *Characidium nambiquara* Zanata & Ohara), all described from tributaries of the Brazilian side of the sub‐basin in the last 6 years and not reported to occur in Bolivia up to date.

Particularly to the Pauserna River, the tributary of the Iténez‐Guaporé River that flows within the boundaries of the Noel Kempff Mercado National Park (NKMNP), a few biodiversity surveys is available (e.g., Killeen et al., 1998). Located in northeastern Bolivia, at the interface of the Amazonian and Cerrado biomes, this park stands out as one of the most biologically diverse and best‐preserved areas in the country and the Amazon basin (Virgilio et al., 2010). Its high conservation status is largely due to its vast extent, ecological heterogeneity and relative isolation from human settlements (IUCN, 2020). Previous ichthyological surveys conducted within NKMNP (i.e., Sarmiento, 1998) reported the presence of *Characidium* sp. (gr. *fasciatum*). Examination of additional material from the region, deposited in the ichthyological collection of the American Museum of Natural History, revealed a new species of *Characidium*, apparently endemic to the park. This study provides the description of the new species and contributes to the knowledge of Bolivian freshwater fish diversity. It also presents novel observations on osteological structures of the Weberian apparatus and offers a brief discussion of the potential geomorphological mechanisms that may have led to the isolation of this species within the Huanchaca Meseta.

## 2 MATERIALS AND METHODS

### 2.1 Ethics statement

**FIGURE 1:**
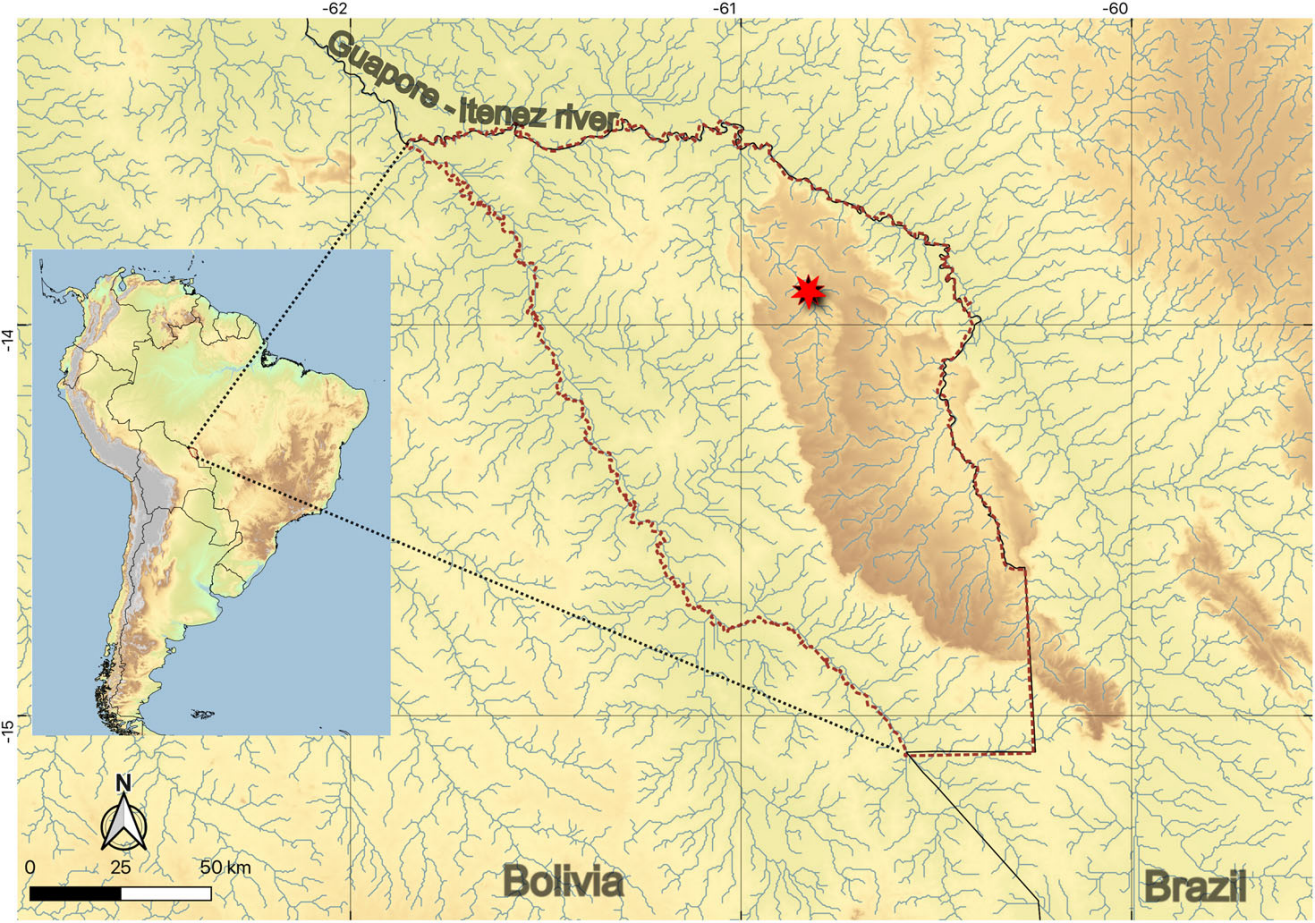
Map representing the Noel Kempff Mercado National Park (red dashed line) and the currently known distribution of the new species; type locality indicated by a red star. The red dashed line represents the boundaries of Noel Kempff Mercado National Park.

The present description was based on specimens collected from the Iténez‐Guaporé River basin, Amazon River basin (Figure 1) by Scott A. Schaefer & Damaris Rodriguez in 1998. This collection was conducted in accordance with the Guidelines for Use of Fishes in Field Research (ASIH, 1987). The collection and exportation of these fishes (permit confirmation number: 273‐2‐1417, on file at Department of Ichthyology, American Museum of Natural History – AMNH) were carried out in accordance with a collaborative agreement established in 1998 between the Museo de Historia Natural ‘Noel Kempff Mercado’ (MNKM) of the Universidad Autónoma Gabriel René Moreno, Santa Cruz de la Sierra and the AMNH.

### 2.2 Morphometric and meristic data collection

Counts and measurements follow Buckup (1993b), Melo and Oyakawa (2015) and Zanata et al. (2023). Measurements are point‐to‐point linear distances taken with digital callipers to a precision of 0.1 mm and expressed as percentages of standard length (*L*_*S*_), except subunits of head, which are given as percentages of head length (*L*_*H*_). In the list of paratypes, asterisks indicate lots for which measurements were included in Table 1. Meristic data are provided in the description; parentheses indicate the number of specimens examined for a particular count, and an asterisk denotes the value for the holotype. Counts of total (abdominal plus caudal) vertebrae, pleural ribs, procurrent caudal‐fin rays and epurals were performed on X‐ray images of the holotype and two paratypes. The patterns of *circuli* and *radii* were observed on scales situated between the dorsal‐fin base and lateral line, after being stained in red alizarin. The pseudotympanum morphology was examined after the removal of the overlying skin, adipose tissue and lateral‐line nerve of alcohol‐preserved specimens. Institutional acronyms follow Fricke and Eschmeyer (2026).

**TABLE 1:** Morphometric data of holotype and paratypes of *Characidium noelkempffi* (*n* = 7), range includes the holotype.

	Holotype	Range	Mean	SD
Total length (mm)	64.2	47.3–66.7	–	–
Standard length (mm)	51.8	37.7–53.7	–	–
Percentages of standard length				
Depth at dorsal‐fin origin	24.5	22.9–25.2	24.1	1.0
Depth at anal‐fin origin	16.6	15.1–17.3	16.2	0.7
Caudal peduncle depth	10.3	9.9–11.5	10.7	0.6
Caudal peduncle length	20.6	17.6–20.6	19.2	0.9
Snout to dorsal‐fin origin	47.0	46.1–48.9	47.2	1.0
Snout to pectoral‐fin origin	24.5	24.2–24.9	24.5	0.3
Snout to pelvic‐fin origin	51.0	50.6–52.3	51.4	0.7
Snout to anal‐fin origin	73.8	73.1–75.4	74.2	0.8
Anal‐apex distance	91.4	91.3–94.3	92.8	1.3
Dorsal‐fin adpressed	25.1	24.7–28.0	26.6	1.3
Pectoral‐fin length	26.2	25.3–26.8	26.4	0.6
Pelvic‐fin length	21.5	20.8–23.6	22.4	1.0
Anus to anal‐fin origin	6.4	5.8–7.1	6.7	0.5
Body width	9.9	9.3–10.7	10.0	0.5
Head length	22.9	22.2–24.5	23.5	0.9
Percentages of head length				
Horizontal eye diameter	26.6	24.5–28.1	26.5	1.1
Snout length	22.0	21.5–23.9	22.6	0.9
Snout to maxillary tip	22.0	21.5–23.9	22.9	0.9
Anterior naris to orbit	9.5	9.5–11.3	10.3	0.7
Posterior naris to orbit	3.5	3.5–5.4	4.5	0.7
Cheek depth	12.0	9.9–12.0	10.9	0.8
Least interorbital width	21.2	18.8–21.4	20.8	0.9

Abbreviation: SD, standard deviation.

### 2.3 CT scan image

Additional osteological data were obtained using micro computed tomography (μCT). Our osteological surveys emphasized characters of the cranium, pectoral girdle and Weberian apparatus. Two paratypes from the same locality as the holotype were scanned using a General Electric Phoenix v|tome|x equipped with a 180 kV Nano Tube, at a voxel resolution ranging from 12.4 to 16.1 μm, with beam energy set at 110 kV and a current of 181 μA, resulting in 1900 projections. The holotype was not scanned to avoid prolonged exposure outside of alcohol, which is required during CT scanning, and to ensure its maximum preservation given its suboptimal condition and the limited number of available specimens. Scanning was conducted at the Microscopy and Imaging Facility of the AMNH; reconstructions were performed using Phoenix datos|x (General Electric, Wunstorf, Germany); datasets were processed using Fiji (Schindelin et al., 2012). The datasets were then opened in 3D Slicer (Fedorov et al., 2012) for segmentation, and the final renderings were edited for presentation using Adobe Photoshop 2024.

Osteological terminology follows Weitzman (1962), with the following modifications suggested by subsequent authors (e.g., Fink & Fink, 1981, 1996; Vari, 1979, 1995; Zanata & Vari, 2005): mesethmoid instead of ethmoid, endopterygoid instead of mesopterygoid, retroarticular instead of articular. We use ventral process of the *os suspensorium* instead of *os suspensorium* and lateral process of the *os suspensorium* instead of rib of fourth vertebra, an adaptation that follows Conway and Britz (2007) and Darlim and Marinho (2018) partially.

## 3 RESULTS

**FIGURE 2:**
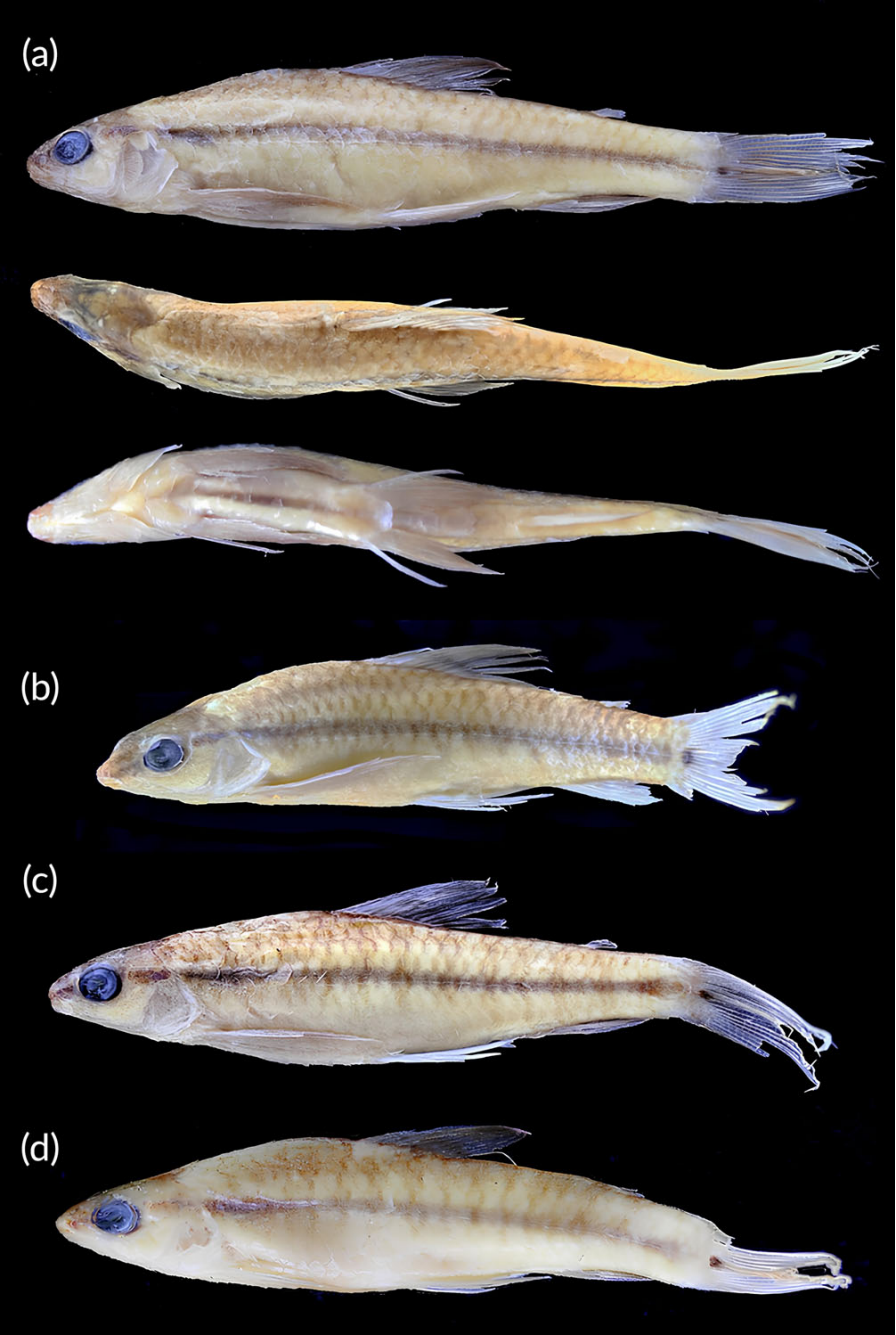
*Characidium noelkempffi*: (a) holotype, American Museum of Natural History (AMNH) 283505, male, 51.8 mm *L*_*S*_, lateral, dorsal and ventral views; (b) paratype, AMNH 229506, male, 39.7 mm *L*_*S*_; (c) paratype, AMNH 229506, female, 40.5 mm *L*_*S*_; (d) paratype, AMNH 229506, female, 42.5 mm *L*_*S*_. All from Bolivia, Santa Cruz, Iténez‐Guaporé River basin, Bolivia, Santa Cruz, Noel Kempff Mercado National Park, Pauserna River.

**FIGURE 3:**
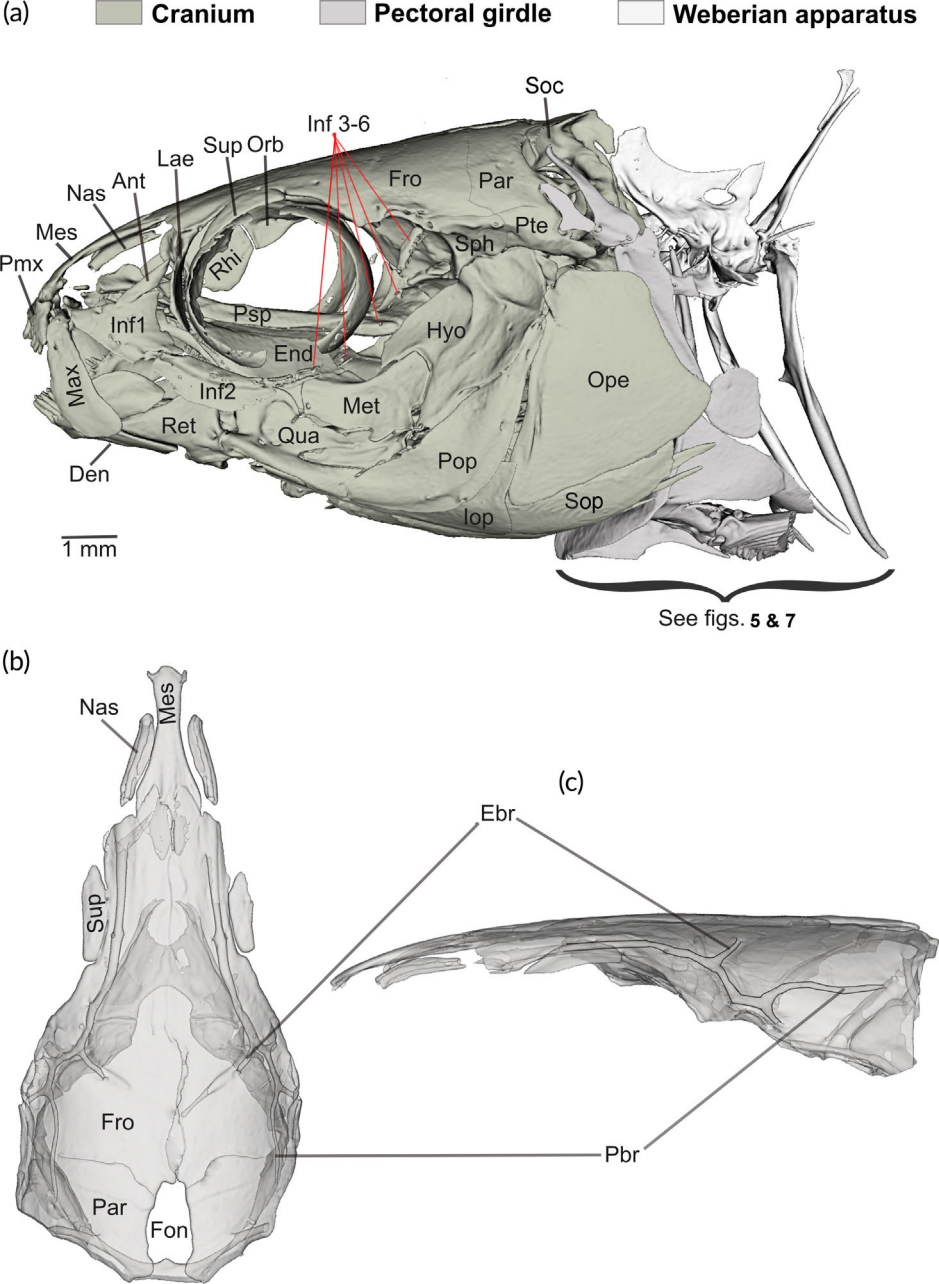
*Characidium noelkempffi*, AMNH 229506, paratype, 53.7 mm *L*_*S*_. (a) Cranium in lateral view; (b, c) neurocranium in dorsal (b) and lateral (c) views, highlighting the supraorbital canal and fontanel positions. Ant, antorbital; Den, dentary; Ebr, epiphysial branch of supraorbital canal; End, endopterygoid; Fon, fontanel; Fro, frontal; Hyo, hyomandibular; Inf 1 to 6, infraorbitals; Iop, interopercle; Lae, lateral ethmoid; Max, maxilar; Mes, mesethmoid; Met, metapterygoid; Nas, nasal bone and nasal canal; Ope, opercle; Orb, orbitosphenoid; Par, parietal; Pbr, parietal branch of supraorbital canal; Pmx, premaxillary; Pop, preopercle; Psp, parasphenoid; Pte, pterotic; Qua, quadrate; Ret, retroarticular; Rhi, rhinosphenoid; Soc, supraoccipital; Sop, subopercle; Sph, sphenotic; Sup, supraorbital.

**FIGURE 4:**
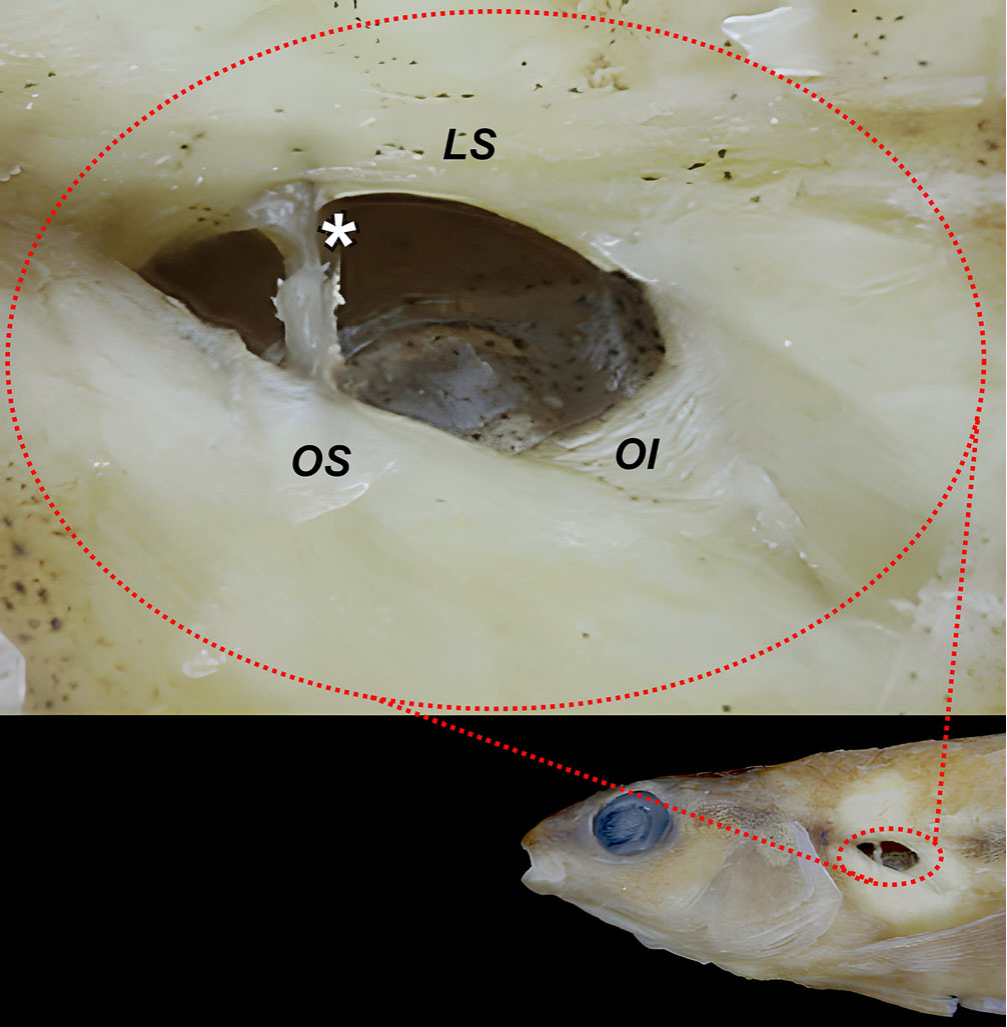
Pseudotympanum of *Characidium noelkempffi* (AMNH 229506, 50.6 mm *L*_*S*_, paratype) in right lateral view. Overlying skin, adipose tissue and lateral‐line nerve removed. (OS) *obliquus superioris*; (OI) *obliquus inferioris*; (LS) *lateralis superficialis*. Asterisk indicates the rib of the fifth vertebra.

**FIGURE 5:**
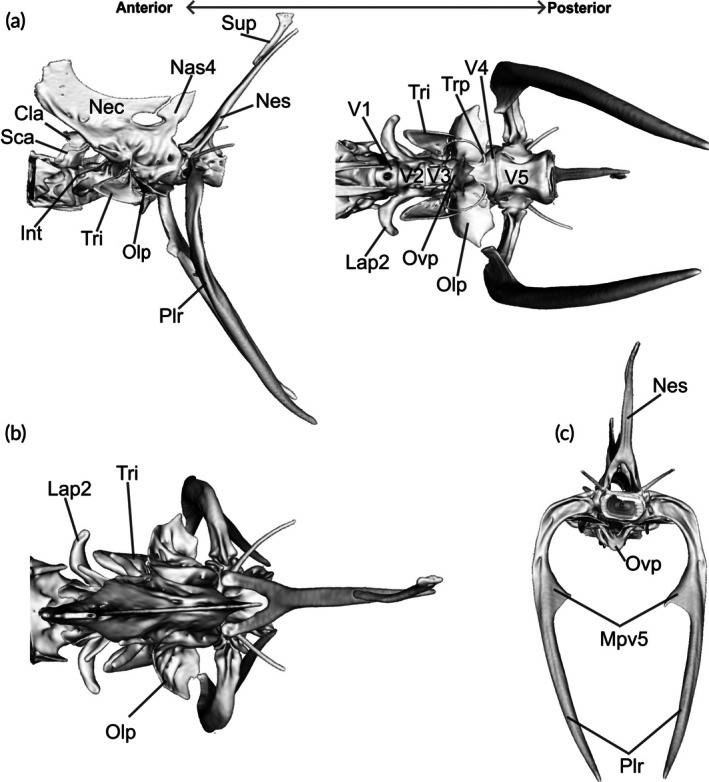
Weberian apparatus of *Characidium noelkempffi*, AMNH 229506, paratype, 53.7 mm *L*_*S*_. (a) Lateral and ventral views; (b) dorsal view; (c) posterior view highlighting the medial process of the fifth vertebra. Cla, claustrum; Int, intercalarium; Lap2, lateral process of centrum 2; Mpv5, medial process of the fifth vertebra; Nas4, neural arch and spine of fourth vertebra; Nec, neural complex; Nes, neural spine; Olp, *os suspensorium* lateral process; Ovp, *os suspensorium* ventral process; Plr, pleural rib; Sca, scaphium; Sup, supraneural; Tri, tripus; Trp, transformator process; V1–V5, vertebra.

**FIGURE 6:**
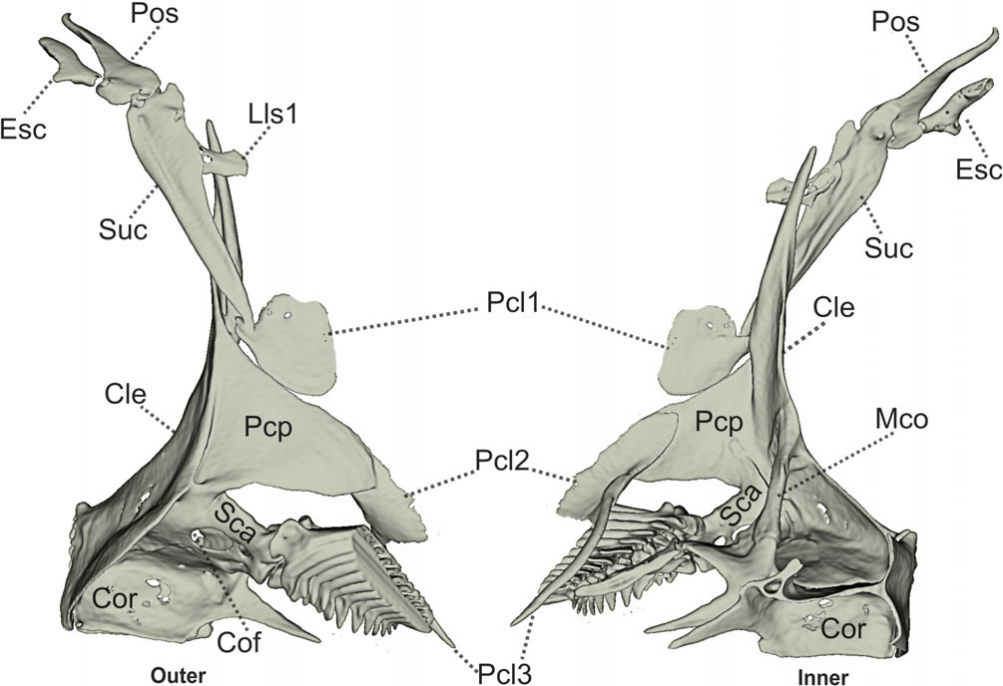
Osteological characteristics of pectoral fin in *Characidium noelkempffi*, AMNH 229506, paratype, 53.7 mm *L*_*S*_. Left pectoral girdle in lateral outer and inner surfaces. Cle, cleithrum; Cof, coracoid foramen; Cor, coracoid; Esc, extrascapular; Lls1, first lateral‐line scale; Mco, mesocoracoid; Pcp, posterior cleithral process; Pcl1, postcleithrum 1; Pcl2, postcleithrum 2; Pcl3, postcleithrum 3; Pos, posttemporal; Sca, scapula; Suc, supracleithrum.

**FIGURE 7:**
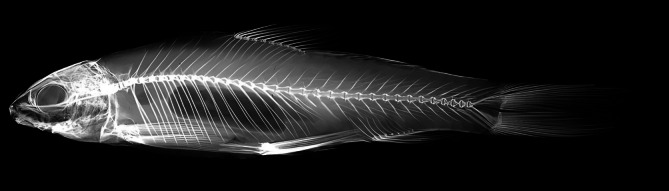
X‐ray of *Characidium noelkempffi*, holotype, American Museum of Natural History (AMNH) 283505, 51.8 mm *L*_*S*_, lateral view.

*Characidium noelkempffi*, new species (Figures 2, 3, 4, 5, 6, 7; Table 1).

urn:lsid:zoobank.org:pub:E17524E4‐DA40‐400E‐821F‐61A02E88B3F8.

urn:lsid:zoobank.org:act:43C116BC‐1FBF‐4EBC‐833B‐00A29A2BA5D2.

### 3.1 Holotype

AMNH 283505, 51.2 mm *L*_*S*_, Bolivia, Santa Cruz, Noel Kempff Mercado National Park, Pauserna River, Meseta Huanchaca I, at mouth of second stream. 13°54′50″ S 60°49′39″ W, 21 August 1998, S. A. Schaefer & D. M. Rodriguez.

### 3.2 Paratypes

All from Bolivia, Santa Cruz, Iténez‐Guaporé River basin, Bolivia, Santa Cruz, Noel Kempff Mercado National Park, Pauserna River. AMNH 229506*, 6, 37.7–53.7 mm *L*_*S*_, collected with holotype. MNKP 3647, 1, 41.6 mm *L*_*S*_, Meseta Huanchaca I, first River below campsite, Pauserna River tributary, 20 Aug 1998, S. A. Schaefer, D. M. Rodriguez. MNKP 3657, 7, 34.2–52.6 mm *L*_*S*_, collected with holotype.

### 3.3 Diagnosis

*Characidium noelkempffi* can be distinguished from all its congeners, except *Characidium lanei* Travassos, *Characidium nana* Mendonça & Netto‐Ferreira, *Characidium samurai* Zanata & Camelier and *Characidium tapuia* Zanata, Ramos & Oliveira‐Silva, by the presence of a broad (at least one scale wide) and conspicuous dark midlateral stripe, markedly darker than the vertical bars (when present), extending continuously from posterior margin of the opercle to the base of the caudal fin, and by the absence of a distinct humeral blotch, either rounded or vertically elongated (Figure 2). The new species can be distinguished from *C*. *lanei* by having four horizontal scale rows above the lateral line (vs. five), 12 circumpeduncular rows of scales (vs. 13 or 14), parietal branch of the supraorbital canal present (vs. parietal branch absent), a clearly visible basicaudal dark spot located somewhat posterior to the end of the longitudinal dark stripe (vs. absence of a clearly visible basicaudal dark spot). *C. noelkempffi* differs from *C*. *nana* by having a complete lateral line (vs. incomplete), presence of adipose fin (vs. absence) and presence of the parietal branch of the supraorbital laterosensory canal (vs. absence). From *C*. *samurai*, the new species differs by having 12 circumpeduncular scale rows (vs. 14), by having a narrower dark lateral stripe, which occupies approximately one scale row (vs. broader, spanning 1.5–2 scale series), and 31–32 pored lateral‐line scales (vs. 34–37). The new species can be distinguished from *C*. *tapuia* by the absence of small dark spot immediately anterior to the insertion of the first dorsal‐fin ray (vs. presence) and, when present, by having 11–16 vertical bars (vs. up to 9 vertical bars). Additionally, *C*. *noelkempffi* can be distinguished from its congeners, except *Characidium dule* Agudelo‐Zamora, Tavera, Murillo & Ortega‐Lara and *C*. *purpuratum*, by having a reddish‐brown hue on the paired, dorsal and caudal fins of majority of the specimens (vs. absence of reddish‐brown hue colouration on fins). The new species can be further distinguished from *C*. *dule* by the presence of postcleithrum 1 (vs. absence) and, when present, by having 11–16 vertical bars (vs. up to 8 vertical bars). From *C*. *purpuratum*, the new species can additionally be diagnosed by the presence of scales on the isthmus (vs. isthmus without scales) and by having five rows of scales below the lateral line (vs. 4). See ‘Discussion’ for additional comments.

### 3.4 Description

Morphometric data of holotype and paratypes are presented in Table 1. Body elongated (Figure 2). Greatest body depth at vertical through dorsal‐fin origin. Dorsal profile convex from tip of snout to naris, and slightly convex to nearly straight from naris to dorsal‐fin origin, almost straight from dorsal‐fin origin to dorsalmost procurrent caudal‐fin ray. Ventral profile of head convex near the dentary symphysis, straight from that point to isthmus, gently convex from that point to pelvic‐fin origin, straight from pelvic‐fin origin to anal‐fin origin and straight to slightly concave from this point to origin of anteriormost ventral procurrent caudal‐fin ray. Snout triangular in lateral view. Mouth subterminal, positioned slightly below ventral margin of the orbit. Distal tip of maxilla not reaching vertical through anterior margin of the orbit. Orbit circular, larger than snout length. Cheek narrow; its depth about one‐quarter to one‐fifth of orbit diameter. Anterior and posterior nares well separated, without raised margins; posterior naris notably closer to orbit than to anterior naris. Supraorbital bone well developed, inner margin slightly convex and outer margin nearly straight (Figure 3). Nasal bones bear a narrow lateral lamella. Parietal fontanel bordered anteriorly by frontals (Figure 3b). Parietal branch of supraorbital canal present (Figure 3c).

Dentary teeth in two rows; outer row with 10(4) or 11*(3) teeth, uni‐ or tricuspid, rarely bicuspid, with lateral cusps distinctly small and hardly visible; teeth decreasing in size from symphysis. Premaxilla with single row of 7(7) teeth similar to those on dentary, decreasing in size from symphysis. Maxillary edentulous. Ectopterygoid with five teeth arranged in one row. Endopterygoid teeth absent.

Scales cycloid; *circuli* absent on exposed portion of scales; up to 11 slightly divergent *radii* present on exposed portion of scales. Lateral line completely pored, with 31(3) or 32*(4) scales; horizontal scale rows above lateral line 4(7); horizontal scale rows below lateral line 5(7). Scales along middorsal line between supraoccipital and origin of dorsal fin 11(6). Scale rows around caudal peduncle 12(7). One (1), 2*(5) or 3(1) scales between anus aperture and anal‐fin insertion. Isthmus and belly completely covered by scales. Pseudotympanum present, limited dorsally by *lateralis superficialis*, anteriorly and posteriorly by *obliquus inferioris* and ventrally by *obliquus superioris*. Humeral hiatus divided into anterior and posterior chambers by pleural rib of fifth vertebra (Figure 4). Paired somewhat leaf‐shaped ventrolateral expansions of fourth centrum, with triangular pointed distal margin and concave surface posterodorsally directed (Figure 5a,b). Each structure formed by relatively thin bone, longer laterally than wide and occupies partially area between transformator process of tripus and vertebral centrum. Both expansions united medially to central ventrally directed and relatively short process, roughly triangular in shape and with rounded distal tip (Figure 5c).

Dorsal‐fin rays ii,8,i*(5) or ii,9,i (2); distal margin of dorsal fin nearly straight or somewhat convex. First dorsal‐fin radial inserts behind 9th(1) or 10th*(2) vertebrae. Adipose fin well developed. Pectoral fin with 11–12 total rays; iii,7,i(3) or iii,8,i*(4); second and third branched pectoral‐fin rays usually longest; posterior tip of pectoral fin reaching or almost reaching pelvic‐fin origin in small specimens (up to 40.0 mm *Ls*) and falling short of pelvic‐fin insertion in larger specimens. Postcleithrum 1, 2 and 3 present (Figure 6). Pelvic‐fin rays i,7,i(7); second to third branched pelvic‐fin rays longest; posterior tip of pelvic fin reaching or not anal‐fin origin. Anal‐fin rays ii,6*(7); posterior margin of anal fin slightly rounded, with second branched ray usually longest. First anal‐fin radial inserts behind 21st(2) or 22nd*(1) vertebra (Figure 7); fin elements on last pterygiophore 2(7). Caudal‐fin rays i,9,8,i(7). Dorsal procurrent caudal‐fin rays 8(3); ventral procurrent caudal‐fin rays 6(3). Total vertebrae 33(2) or 34*(1). Supraneural bones 4(2) or 5*(1). Epural bones 2(3). Uroneural bone 1(3) (Figure 7).

### 3.5 Colour in alcohol

Ground colour of head and body light yellowish. Dorsal surface of head darker than laterals and remaining portions of the body; difference more evident in dorsal view of males (Figure 2a). In lateral view, dorsal half of head not distinctly darker than ventral half; a conspicuous and narrow dark stripe extends from tip of snout to eye, aligned with dark stripe posterior to eye to end of dorsal portion of opercle. Ventral half of head with small melanophores homogeneously distributed over cheek, infraorbitals and opercle. Ventral surface of head with similarly distributed tinny melanophores, more concentrated on laterals and with clearer roughly triangular central area. Dark humeral blotch absent. Midlateral dark stripe conspicuous, relatively broad (approximately one scale wide), extending from the rear of opercle to end of caudal peduncle; portion of stripe anterior to dorsal‐fin origin running over scale row immediately above lateral line and shifting to over lateral‐line row on posterior part of body. Midlateral stripe apparently with straight borders overall, but with somewhat waved borders in some stretches (seen under stereomicroscope). Posterior end of midlateral stripe somewhat diffuse and slightly expanded, but not conforming a distinct peduncular blotch. Black basicaudal spot conspicuous, rounded, located posterior to termination of midlateral stripe, not continuous to it. Males and females with distinct pattern of colouration on flanks, regarding occurrence of bars. Females possess 11–16 dark vertical bars, regularly spaced, distributed from the rear of head to end of caudal peduncle; bars usually formed by melanophores concentrated on posterior portion of scales, resulting in a chain‐like appearance. Bars more evident above midlateral stripe, slightly trespassing it ventrally on anterior portion of body and reaching ventral portion on posterior third of body, especially posterior to the anal‐fin origin (Figure 2c). Males without dark bars; dorsal half of body with melanophores concentrated near the margins of scales, resulting in a reticulate pattern (Figure 2b); two or three dorsalmost horizontal series of scales with stronger concentration of melanophores on posterior portion of scales, forming inconspicuous horizontal series of vertically elongated blotches (Figure 2a); smaller males (<40 mm *L*_*S*_) with faint concentration of melanophores ventrally to midlateral stripe, possibly representing remnants of bars (Figure 2b). Ventral surface of body with tinny dark melanophores overall. Paired fins, dorsal and central most caudal‐fin rays with a reddish‐brown hue, apparently more evident on males. Dark hue mainly on borders of rays on pectoral and pelvic fins, and on borders of rays and interadial membranes on dorsal and caudal fins. A dark inconspicuous oblique band positioned below midlength of dorsal fin, extends from unbranched rays to last branched rays, formed mainly by melanophores concentrated on area of membranes close to laterals of rays. Other fins without dark bands. Basicaudal spot well defined. Anal fin of females mostly hyaline; anal fin of males with melanophores on borders of rays and membranes between ramifications of rays. Adipose fin with scattered tinny melanophores.

### 3.6 Sexual dimorphism

Hooks on fins absent. Sexual dichromatism evident on body flanks: adult males lacking vertical bars (Figure 2a), females with 11–16 vertical bars, variable in shape and extension onto the ventral region (Figure 2c). Males also showed dark pattern of colouration on fins more evident. For additional details, see Colouration in alcohol.

### 3.7 Distribution

*C. noelkempffi* is only known from tributaries of the Pauserna River, a tributary of the Iténez–Guaporé River basin, within Noel Kempff Mercado National Park, Santa Cruz, Bolivia, a tributary of the Madeira River, Amazon basin. All specimens were collected on the Meseta Huanchaca I, an isolated sandstone plateau where altitudes range approximately between 500 and 900 m a.s.l. (Figure 1).

### 3.8 Conservation status

*Characidium noelkempffi* is currently known only from its type locality, a small tributary of the Río Pauserna within Parque Nacional Noel Kempff Mercado, Bolivia. The species was collected over 25 years ago during biological surveys, and no additional specimens have been obtained since the original collection. The extreme remoteness of the type locality, combined with logistical challenges and security concerns that have historically affected access to certain areas of the region, has prevented subsequent surveys to assess population status. The species' apparent restriction to a single small stream system within a well‐protected national park provides substantial habitat protection, as NKMNP is recognized as a UNESCO World Heritage Site with relatively intact ecosystems and effective conservation management (Killeen et al., 1998). However, the species' apparent endemism to a specific sub‐basin makes it inherently vulnerable to any potential future environmental changes, as documented for other headwater‐endemic Characiformes in isolated drainage systems (Melo et al., 2021; Nogueira et al., 2010; Oliveira‐Silva et al., 2022). Following IUCN Red List criteria (IUCN Standards and Petitions Committee, 2024), *C. noelkempffi* qualifies as data deficient (DD) due to insufficient information regarding its distribution, population size and ecological requirements. Given its occurrence within a well‐protected area, the conservation outlook appears favourable, though priority should be given to future biological inventories in accessible portions of the Pauserna drainage system. The species' discovery highlights the continued importance of biodiversity documentation in remote Neotropical protected areas, where many endemic taxa likely await scientific discovery within relatively secure conservation frameworks.

### 3.9 Etymology

The specific name *noelkempffi* is dedicated to Professor Noel Kempff Mercado (1924–1986), a Bolivian biologist and environmentalist renowned for his contributions to the study and conservation of Bolivia's biodiversity. As a professor at the Universidad Autónoma Gabriel René Moreno, director of the Santa Cruz Botanical Garden and Zoo and member of the Bolivian Academy of Sciences, Kempff Mercado played a pivotal role in promoting environmental research in Bolivia (Killeen et al., 1998). His extensive work included publications on Bolivian herpetofauna, Amazonian flora and avian species. Professor Kempff Mercado was tragically killed on 5 September 1986 during a biological expedition in the Serranía de Caparuch when his research team inadvertently encountered a clandestine narcotics operation (Steinberg, 1998). In recognition of his legacy, the Huanchaca National Park was renamed Parque Nacional Noel Kempff Mercado in 1988 (Decreto Supremo No.: 21997, 1988). The naming of this species honours his lifelong dedication to the exploration and preservation of Bolivia's natural heritage.

## 4 DISCUSSION

The discovery of *C. noelkempffi* adds to the still limited knowledge of Crenuchidae diversity in Bolivian tributaries of the Madeira River basin, a region where taxonomic studies on this group remain relatively scarce. The characterization of the new species contributes not only to a more accurate inventory of the regional fauna but also provides an opportunity to refine our understanding of diagnostic characters within the genus, particularly with respect to body colouration, the occurrence of sexual dichromatism and osteological features. As indicated previously, eight congeners of the new species are reported to occur in Bolivian stretches of rivers draining to the Madeira River basin (i.e., *C. bolivianum*, *C. fasciatum*, *C. heinianum*, *C. laterale, C. purpuratum*, *C. schindleri*, *C. steindachneri* and *C. zebra*). *C. noelkempffi* can be promptly differentiated from *C. bolivianum*, *C. fasciatum* and *C. purpuratum* by having the isthmus completely covered by scales (vs. naked isthmus), from *C. laterale* by having a complete lateral line and absence of a caudal peduncle blotch (vs. lateral line incomplete and presence of a caudal peduncle blotch), from *C. heinianum* by the overall robust body and absence of thin dark bars on flanks of both sexes (vs. elongated narrow body and presence of 16–23 thin vertical dark bars) and from *C. schindleri* by the absence of conspicuous dark blotches along midlateral portion of body and by having four scale rows above the lateral line (vs. presence of midlateral blotches and five scale rows above the lateral line). Among the species listed to occur in the tributaries of the Madeira River draining Bolivia, *C*. *noelkempffi* shares the overall body colouration and an isthmus covered with scales with *C. steindachneri* and *C. zebra*. The new species differs from both species by having 12 circumpeduncular scale rows (vs. 14) and by its colouration, mainly by the presence of a conspicuous dark lateral stripe (at least one scale wide) extending from tip of snout to end of caudal peduncle (vs. stripe absent or inconspicuous), and absence of dark vertical bars in adult males (vs. presence).

Although most antique descriptions of species in *Characidium* lack information about colouration variation within a species, more recent descriptions have revealed that such variation is quite common. Particularly, sexual dichromatism has often been reported as variation in pigmentation intensity between males and females of the genus, occurring in *Characidium bimaculatum*, *Characidium pumarinri*, *Characidium orientale*, *Characidium stigmosum*, *Characidium vestigipinne*, *Characidium satoi* and *Characidium mirim* (Buckup & Hahn, 2000; Buckup & Reis, 1997; Melo & Buckup, 2002; Melo & Espindola, 2016; Melo & Oyakawa, 2015; Netto‐Ferreira et al., 2013; Teixeira & Melo, 2020). The sexual dichromatism of *C. noelkempffi* is distinct from the cited congeners, except for *C. bimaculatum*, by the complete absence of dark vertical bars in adult males while present in females. Although a similar condition was reported in males of *C. bimaculatum* by Melo and Espindola (2016), both species differ markedly in other sexually dimorphic traits and morphological features. Males of *C. bimaculatum* possess bony processes on the branched pelvic‐ and pectoral‐fin rays, which are apparently absent in *C. noelkempffi*. Additionally, *C. bimaculatum* possesses 14 circumpeduncular scales (vs. 12 in *C. noelkempffi*) and presents a conspicuous rounded blotch on the caudal peduncle, absent in the new species.

### 4.1 The Weberian apparatus in *Characidium*, with a shell‐like structure newly described to the genus

The Weberian apparatus represents one of the most remarkable morphological innovations among Ostariophysan fishes, consisting of a series of highly modified anterior vertebrae that establish a functional connection between the swim bladder and the auditory system (Alexander, 1962; Grande & Young, 2004). This structure, composed of Weberian ossicles derived from modified vertebrae, enhances auditory sensitivity by transmitting and amplifying sound waves detected by the swim bladder to the membranous labyrinth of the inner ear (Alexander, 1962; Ladich & Popper, 2004). Somewhat detailed investigations have documented substantial variation in the ossicles of the Weberian apparatus, vertebral modifications and associated structures in taxa of some Ostariophysan lineages, such as Cypriniformes and Siluriformes (e.g., Bird et al., 2020; Duclos & Grande, 2025). Contrastingly, despite its recognized phylogenetic and functional significance, detailed osteological descriptions of the Weberian apparatus remain scarce for several groups of Characiformes, particularly within the family Crenuchidae. The broadest treatment prior to the present contribution is that of Buckup (1993a), who examined variation in some Weberian structures and employed them as characters in his phylogenetic reconstruction of Characidiinae. For instance, the author reported differences in the degree of fusion among the anterior Weberian centra, with all four centra forming a single osseous complex in *Melanocharacidium pectoral* Buckup and *Melanocharacidium depressum* Buckup, whereas in *Characidium declivirostre* Steindachner only centra 3–4 (and possibly centrum 2) are fused, whereas in the remaining species analysed the centra were described as free. Additional characters included the presence or absence of ventral processes on centrum 2 and the presence of a medial process on the rib of the fifth centrum. Although informative for phylogenetic purposes, these observations did not encompass the full range of osteological elements that compose the Weberian apparatus. A few recent studies, focused on the description of species of *Characidium*, provided some information about the variation in the swim bladder attachment to the Weberian apparatus along with remarkable variation in swim bladder size and shape (e.g., Agudelo‐Zamora, Ortega‐Lara, & Donald, 2020a; Agudelo‐Zamora, Tavera, et al., 2020b; Zanata et al., 2020). Armbruster et al. (2021), in accordance with Buckup (1993a), provided more information on the fusion of vertebral centra of the Weberian apparatus in some fast‐flowing‐water species of *Characidium*. The authors reported fusion of vertebral centra 2 + 3 in *Characidium crandellii* Steindachner, and fusion centra 2–4 in *Characidium declivirostre*, *Characidium duplicatum* Armbruster, Lujan & Bloom and *Characidium wangyapoik* Armbruster, Lujan & Bloom. In addition, Armbruster et al. (2021) described an expansion of the fourth centrum in all cited species, extending anteriorly towards the lateral process of second centrum.

As given in the description, the examination of CT scan images of *C. noelkempffi* revealed the existence of a paired remarkable shell‐like and somewhat leaf‐shaped ventrolateral expansion of the fourth centrum (Figure 5a,b). Somewhat similar expansions of the fourth centrum were illustrated to the small characid *Moenkhausia lepidura* by Darlim and Marinho (2018). The authors followed Conway and Britz (2007) and named the lateral expansions as outer arms of the *os suspensorium* and the ventral process projecting ventrally as inner arm (their Figure 4b OsSo and OsSi, respectively). Although a developmental study of the formation of the Weberian ossicles was not performed herein, we followed the cited authors in considering the ventrolateral and ventral expansions of the fourth centrum of *C. noelkempffi* as modifications of the *os suspensorium*. However, we prefer to nominate the expansions as processes instead of arms, and as lateral and ventral processes, due to the markedly different format of the structures seen in C. *noelkempffi* compared to that of the small cyprinid *Sundadanio axelrodi* (Brittan) illustrated by Conway and Britz (2007: 1564, Figure 1). As stated previously, particularly to *Characidium*, Armbruster et al. (2021) registered an expansion of the fourth centrum to *C. crandellii*, *C. declivirostre, C. duplicatum* and *C. wangyapoik*, a structure apparently not similar to that of C. *noelkempffi*. According to the authors, the four species examined by them possess ‘vertebral centra 2 + 3 fused, without ventral processes’ and ‘rib of centrum 4 distally expanded, extending anteriorly towards lateral process of centrum 2’. In C. *noelkempffi*, no fusion of centra was observed, nor was their an anterior extension to the second centrum.

The present study provides the first CT scan images to members of Crenuchidae, along with new description of Webberian process revealing a degree of structural detail not previously reported for this group. These data contribute to advance the morphological knowledge of Crenuchidae and establish a foundation for future comparative studies within the genus *Characidium*. A more detailed comprehensible comparative analysis, including variation in the ossicles of the Weberian apparatus, vertebral modifications and associated structures within *Characidium*, is part of ongoing projects led by two of the authors (A.M.Z and L.O.S).

### 4.2 Geomorphological context and implications for isolation

*C. noelkempffi* was collected on the upland streams of the Huanchaca Meseta (Meseta Huanchaca I), within Noel Kempff Mercado National Park, in the headwaters of the Paucerna River (Figure 1). The geomorphological context of this plateau is presented here to explain the isolation of the new species and the origin of the dispersal barriers that delimit its restricted distribution. The plateau was built upon sandstones and quartzites of the Brazilian Shield, with deposition and lithification occurring approximately 1.1 billion years ago during the Mesoproterozoic (Cohen et al., 2025; Litherland & Power, 1989). According to the first authors, following this ancient basement formation, subsequent geological events reshaped the landscape into its modern form. During the Mesozoic, localized sedimentary cover (including Cretaceous sandstones) was deposited above the older units. In the Cenozoic, particularly during the Neogene (Miocene–Pliocene), regional tectonic uplift, fault reactivation and cycles of erosion and peneplanation (partially influenced by the Andean orogeny and associated flexural stresses) further modified the plateau surface, producing mesa tops capped by duricrusts and steep escarpments (Lamb, 2016). Quaternary climatic oscillations influenced drainage networks, altering stream incision and waterfall development, whereas Pleistocene–Holocene vegetation and hydrological changes likely reinforced the isolation of headwater streams and aquatic habitats on the plateau (Burbridge et al., 2004; Maezumi et al., 2015; Mayle et al., 2000). These combined processes (i.e., Mesoproterozoic basement formation, Neogene tectonic uplift, erosion cycles and Quaternary climate/hydrology) created the pronounced relief and hydrological discontinuities that are central to understanding the isolation of freshwater biota in the Huanchaca Meseta.

Although the Meseta's bedrock predates the evolutionary history of *Characidium*, whose diversification began roughly ~50 million years ago (Poveda‐Martínez et al., 2016), these more recent tectonic and climatic events plausibly acted as isolating mechanisms during the genus' evolutionary history. Recurrent uplift and erosion during the Neogene, combined with Quaternary hydrological shifts and waterfall formation, likely fragmented river networks and created persistent high‐elevation refugia for freshwater biota. Streams descending from the Huanchaca Plateau towards the Pauserna and Verde rivers form numerous cascades and escarpments along the eastern margin of the Serranía. These geomorphological features, including the Arco Iris (Rainbow) and the downstream Frederico Ahlfeld Falls, produce marked altitudinal discontinuities between the plateau surface (commonly between ~600–900 m a.s.l.) and the surrounding lowlands (~700–750 m a.s.l.) (Litherland & Power, 1989; Torres et al., 1999). Published accounts and park descriptions (e.g., Sobre Libros y Cultura, 2024) report Arco Iris at ~88 m and Frederico Ahlfeld at approximately 45 m. Such cascades and steep escarpments constitute effective dispersal barriers for small benthic fishes (e.g., *Characidium*). Notably, the newly described species is restricted to the upper reaches of the Pauserna drainage, occurring above these waterfalls, a distribution consistent with long‐term isolation caused by the plateau's escarpment and its associated cascades (Torres et al., 1999). This scenario is reinforced by the observation that the new species lacks the morphological adaptations found in other *Characidium* species that can traverse small waterfalls, such as more robust pectoral fins used to cling to rocks, as described in *Characidium iaquira* Zanata, Ohara, Oyakawa & Dagosta and related taxa (Zanata et al., 2020). The absence of such adaptations in the new species likely limits its ability to overcome such natural barriers, supporting its long‐term isolation in high‐elevation headwater streams.

Comparable patterns of range restriction and lineage divergence linked to upland plateaus and escarpment waterfalls have been documented for other freshwater fishes in shield and highland systems. Narrow endemics and isolated populations occur in Guiana Shield drainages above major waterfalls (Armbruster et al., 2021; Lujan et al., 2013), and phylogeographic analyses of loricariid catfishes reveal strong genetic structuring and endemic radiations associated with plateau headwaters (Lujan et al., 2020). Empirical studies demonstrate that waterfalls and cascades are significant barriers shaping fish assemblages (Barbosa et al., 2015), whereas regional modelling confirms that Neogene–Quaternary landscape reorganization, including uplift, river capture and climate oscillations, has been a recurrent driver of allopatric diversification in South American fishes (Cassemiro et al., 2023; Oliveira‐Silva et al., 2023). The ‘islands‐in‐the‐sky’ dynamic proposed for tepui biotas (e.g., Kok, 2013) provides an apt analogue for aquatic systems of the Huanchaca Meseta, where escarpment isolation and plateau‐top persistence promote long‐term endemism. Thus, the discovery of a morphologically distinct and range‐restricted *Characidium* lineage on Meseta Huanchaca likely reflects the combined influence of Neogene tectonic uplift, Quaternary climatic variability and the consequent formation of persistent high‐elevation hydrological isolates within an ancient geological framework. Future studies incorporating broader spatial and taxonomic sampling within the Huanchaca Meseta, including other *Characidium* species, and integrating phylogenetic and biogeographic approaches, will be essential to test these hypotheses regarding diversification in this specific highland aquatic system. Such studies will provide a more comprehensive understanding of the evolutionary history, patterns of endemism and the mechanisms driving diversification in isolated upland streams, with *Characidium* serving as an excellent model for these investigations.

## 5 COMPARATIVE MATERIAL EXAMINED

Comparative material was obtained from the list of species provided by Zanata et al. (2018, 2023) and Oliveira‐Silva et al. (2026).

## AUTHOR CONTRIBUTIONS

Conceptualization: Leonardo Oliveira‐Silva and Scott A. Schaefer. Developing methods: Leonardo Oliveira‐Silva and Angela Zanata. Conducting the research, data analysis, data interpretation, writing and funding: all authors.

## FUNDING INFORMATION

This work was financed by the São Paulo Research Foundation (FAPESP; postdoctoral fellowship grant 2023/09871‐6 and BEPE – *Bolsa Estágio de Pesquisa no Exterior* grant 2024/16565‐1 to Leonardo Oliveira‐Silva).

## CONFLICT OF INTEREST STATEMENT

This study was conducted without any conflict of interest.

## Data Availability

Data will be made available upon acceptance.
